# Synergistic Combinations of Curcumin, Sulforaphane, and Dihydrocaffeic Acid against Human Colon Cancer Cells

**DOI:** 10.3390/ijms21093108

**Published:** 2020-04-28

**Authors:** Jesús Santana-Gálvez, Javier Villela-Castrejón, Sergio O. Serna-Saldívar, Luis Cisneros-Zevallos, Daniel A. Jacobo-Velázquez

**Affiliations:** 1 Tecnologico de Monterrey, Escuela de Ingeniería y Ciencias, Ave. Eugenio Garza Sada 2501, Monterrey, NL C.P. 64849, Mexico; 2 Department of Horticultural Sciences, Texas A&M University, College Station, TX 77843-2133, USA

**Keywords:** nutraceutical combinations, synergism, colon cancer, combination index, MTS assay

## Abstract

Nutraceutical combinations that act synergistically could be a powerful solution against colon cancer, which is the second deadliest malignancy worldwide. In this study, curcumin (C), sulforaphane (S), and dihydrocaffeic acid (D, a chlorogenic acid metabolite) were evaluated, individually and in different combinations, over the viability of HT-29 and Caco-2 colon cancer cells, and compared against healthy fetal human colon (FHC) cells. The cytotoxic concentrations to kill 50%, 75%, and 90% of the cells (CC_50_, CC_75_, and CC_90_) were obtained, using the MTS assay. Synergistic, additive, and antagonistic effects were determined by using the combination index (CI) method. The 1:1 combination of S and D exerted synergistic effects against HT-29 at 90% cytotoxicity level (doses 90:90 µM), whereas CD(1:4) was synergistic at all cytotoxicity levels (9:36–34:136 µM) and CD(9:2) at 90% (108:24 µM) against Caco-2 cells. SD(1:1) was significantly more cytotoxic for cancer cells than healthy cells, while CD(1:4) and CD(9:2) were similarly or more cytotoxic for healthy cells. Therefore, the SD(1:1) combination was chosen as the best. A model explaining SD(1:1) synergy is proposed. SD(1:1) can be used as a basis to develop advanced food products for the prevention/co-treatment of colon cancer.

## 1. Introduction

Cancer, a chronic disease, is the second leading cause of death worldwide, accounting for millions of fatalities every year [1]. Nutraceuticals are promising compounds naturally found in foods that have preventive and therapeutic properties against chronic diseases. Hence, foods rich in nutraceuticals may be an alternative for cancer prevention and co-treatment. However, no single nutraceutical or food has been found to be completely effective against cancer. This could be due to the fact that cancer is a multifactorial disease, as it can be caused by several intrinsic factors (e.g., inherited mutations, hormones, immune conditions, and oxidative stress) and some environmental or extrinsic factors (e.g., tobacco, diet, radiation, and infectious organisms) [2]. Nutraceutical combinations could be a powerful solution, as they can act synergistically against chronic diseases, i.e., an effect greater than the sum of the effects of the individual compounds, by attacking different targets of the disease. Moreover, the combination of phytochemicals can interfere or activate different cell mechanisms by improving the bioavailability of active compounds, which leads to dose reduction and thereby a decrease of toxic effects, among other advantages [2]. Nutraceutical combinations can be used, therefore, to formulate novel and highly effective food products for the prevention and co-treatment of chronic diseases, including cancer.

Recently, we proposed a method for designing advanced food products against chronic diseases [2]. Briefly, it consists of the following sequential steps: (1) selecting the chronic disease; (2) selecting the nutraceutical candidates; (3) performing combination in vitro studies; (4) determining the best combination; (5) selecting food ingredients that are rich in the nutraceuticals of the best combination; (6) performing in vitro gastrointestinal digestion studies of the ingredients, to determine the bioavailability of the nutraceuticals; (7) performing combination studies of the digested ingredients based on the doses and proportions of the best combination, and if necessary, testing other combinations; (8) using the new information to design a highly effective food, beverage, or supplement for the prevention/co-treatment of the selected chronic disease; (9) testing in laboratory animals; (10) testing in clinical human trials; and (11) performing shelf-life studies. In the present study, we are undertaking the first four steps of this methodology, to find advanced nutraceutical combinations against colon cancer.

Colon cancer is the third most common (1.8 million cases) and the second death-causing cancer (over 800,000 deaths) [1]. Only 5–10% of colon cancers are due to genetic predisposition, while most of them are attributed to diet and lifestyle habits [3]. In addition, cancer is a multitarget disease, meaning that it can be attacked in several ways, such as inhibiting cell proliferation; inducing cell differentiation and apoptosis; and deterring angiogenesis and metastasis [4,5]. For these reasons, colon cancer is an excellent candidate for designing highly effective food products, using our proposed method.

Some nutraceuticals that have activity against colon cancer include curcumin, sulforaphane, and chlorogenic acid. Curcumin is a diphenolic compound and a yellow pigment from turmeric (*Curcuma longa*), the root of an Asian spice. Curcumin has been found to cause cell-cycle arrest, apoptosis, and invasion repression in different colon cancer cell lines [6,7,8]. In rodents, curcumin has shown beneficial effects against neoplastic lesions, macroscopic tumors, and aberrant crypt foci (ACF) [9,10]. ACF reduction has also been observed in humans [11]. Since curcumin is poorly absorbed in the human gastrointestinal tract, it has been recognized as an advantage for the management of colon cancer [11].

Sulforaphane is an isothiocyanate abundant in cruciferous vegetables [12]. Sulforaphane has inhibited the viability, proliferation, and angiogenesis of colon cancer cells [13,14,15]. Significant reduction of total ACF, multicrypt foci, and mean tumor weight has also been achieved in animals [16,17]. Normally, sulforaphane is in the form of glucoraphanin, a glucosinolate; however, when the vegetable is macerated or eaten, the enzyme myrosinase hydrolyzes glucoraphanin to sulforaphane [12]. In the small intestine, sulforaphane is much better absorbed than glucoraphanin [18,19]. Cooking inactivates myrosinase, resulting in the ingestion of intact glucoraphanin. Then, glucoraphanin arrives into the colon and can be hydrolyzed to sulforaphane by the microbiota with thioglucosidase activity [12]. Thus, sulforaphane is another nutraceutical candidate against colon cancer.

Chlorogenic acid is one of the most studied phenolic compounds, widely distributed in plant foods [20]. Chlorogenic acid has also shown cytotoxic activity in colon cancer cells [21] and antitumoral activity in murine models [22]. However, chlorogenic acid is extensively metabolized by the human body, which may alter the activity observed in vitro, while the effects found in vivo could be caused by its metabolites. About 1/3 of the ingested chlorogenic acid is readily absorbed in the small intestine [23] and further decomposed by the body, while the rest reaches the colon, where it is further metabolized by the colonic microbiome [24,25,26]. Dihydrocaffeic acid (3,4-dihydroxyhydrocinnamic acid, 3-(3,4-dihydroxyphenyl)propionic acid, hydrocaffeic acid) is one of the main metabolites of chlorogenic acid produced by the colonic microbiota. The anticancer potential of dihydrocaffeic acid against human cancer cell lines MCF-7 (breast), PC-3 (prostate), and HCT-116 (colon) has been recently reported [27]. Therefore, it is a relevant candidate for cancer prevention and treatment. 

Nutraceutical combinations that have shown more potent effects than the individual compounds against colon cancer in vitro include sulforaphane and diindolylmethane [28]; indole-3-carbinol and genistein [29]; and polydatin and resveratrol [30]. Moreover, the combinations that have shown more potent effects in animals include curcumin and green tea catechins [31]; curcumin and epigallocatechin-3-gallate [32]; curcumin and resveratrol [33]; sulforaphane and dibenzoylmethane [34]; and diindolylmethane and butyrate [35]. Meanwhile, in humans, it has been reported that the combination of curcumin and quercetin exerted positive effects, but it was not established whether the combinations were more effective than the dosing of individual compounds [36]. 

The present study was undertaken to evaluate different combinations of curcumin, sulforaphane, and dihydrocaffeic acid over the viability of two different types of colon cancer cells, to find the best combination that can be further used to formulate advanced food products with anticancer properties.

## 2. Results

### 2.1. Validation of Dose-Effect Data of Curcumin, Sulforaphane, Dihydrocaffeic Acid and Their Constant-Ratio Combinations

The r-value of the linearized dose-effect curves (Equation (2), shown in Section 4.3 of Materials and Methods) for curcumin, sulforaphane, dihydrocaffeic acid, and their constant-ratio combinations are depicted in Table 1. All compounds and combinations had r-values > 0.95 for both HT-29 and Caco-2 cancer cells; hence, the data were good enough to determine the type of effect (synergistic, additive, or antagonistic) by using the combination index (CI) method.

### 2.2. Effects of Equimolar Combinations of Curcumin, Sulforaphane, and Dihydrocaffeic Acid on the Viability of HT-29 Colon Cancer Cells

**Figure 1:**
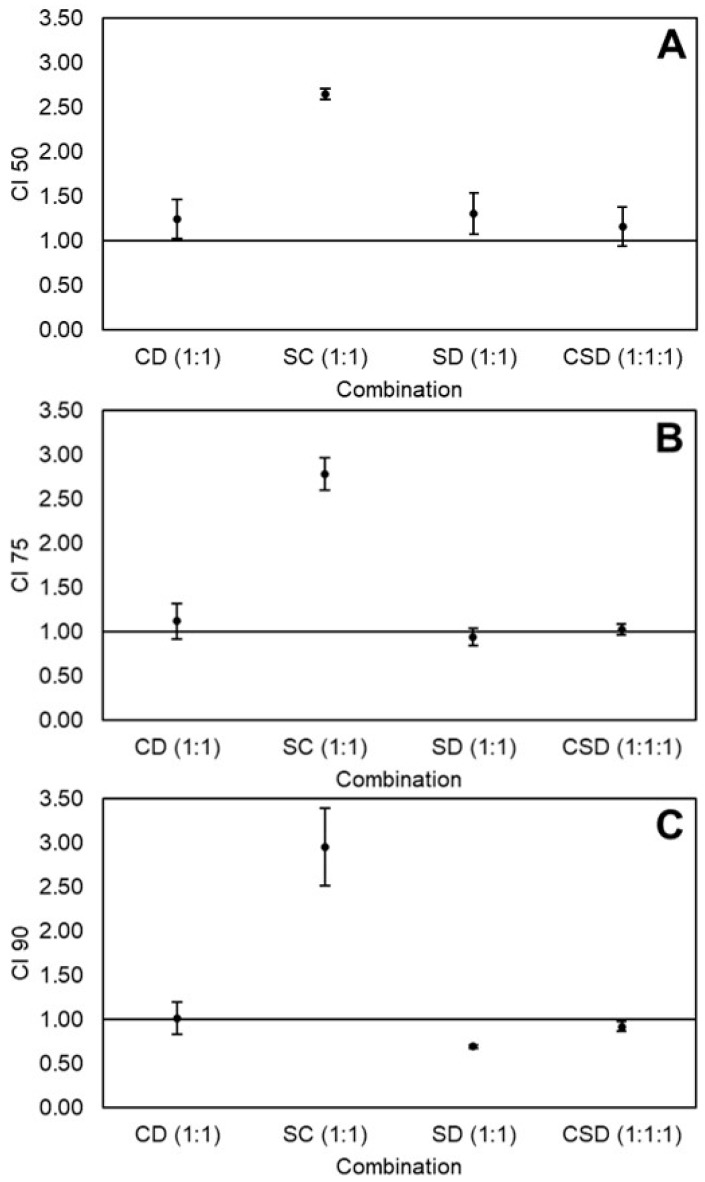
Combination index (CI) values of equimolar combinations of curcumin (C), sulforaphane (S), and dihydrocaffeic acid (D) on the viability of HT-29 colon cancer cells at 50% (**A**), 75% (**B**), and 90% (**C**) cytotoxicity levels. Values represent the mean ± standard error of two independent experiments performed on different days, with each experiment having three replicates. Abbreviations: CI 50, 75, and 90 = Combination index at 50%, 75%, and 90% cytotoxicity.

Dose-effect data of curcumin, sulforaphane, dihydrocaffeic acid, and the equimolar combinations curcumin-dihydrocaffeic acid(1:1) [CD(1:1)], sulforaphane-curcumin(1:1) [SC(1:1)], sulforaphane-dihydrocaffeic acid(1:1) [SD(1:1)], and curcumin-sulforaphane-dihydrocaffeic acid [CSD(1:1:1)] were analyzed, to determine the effect on the viability of HT-29 cells, by calculating the CI values at 50%, 75%, and 90% cytotoxicity level (Figure 1A–C, respectively). At 50% and 75% cytotoxicity, the effects of CD, SD, and CSD were nearly additive (CI ≈ 1), while SC showed a relatively high antagonistic effect (CI between 2.5 and 3). However, at 90% cytotoxicity, SD exerted a significant synergistic effect (CI ≈ 0.70). The doses of the individual nutraceuticals, as well as the doses and dose-reduction index (DRI) values of the combinations at all cytotoxicity levels, are shown in Table 2. The synergistic combination SD at 90% cytotoxicity caused a 2x dose reduction of sulforaphane and 10x of dihydrocaffeic acid, whereas SC at all cytotoxicity levels showed a DRI < 1, which means that the combination caused an increase in the dose needed to kill the cancer cells, making it antagonistic. 

### 2.3. Effects of Equimolar Combinations of Curcumin, Sulforaphane, and Dihydrocaffeic Acid on the Viability of Caco-2 Colon Cancer Cells

**Figure 2:**
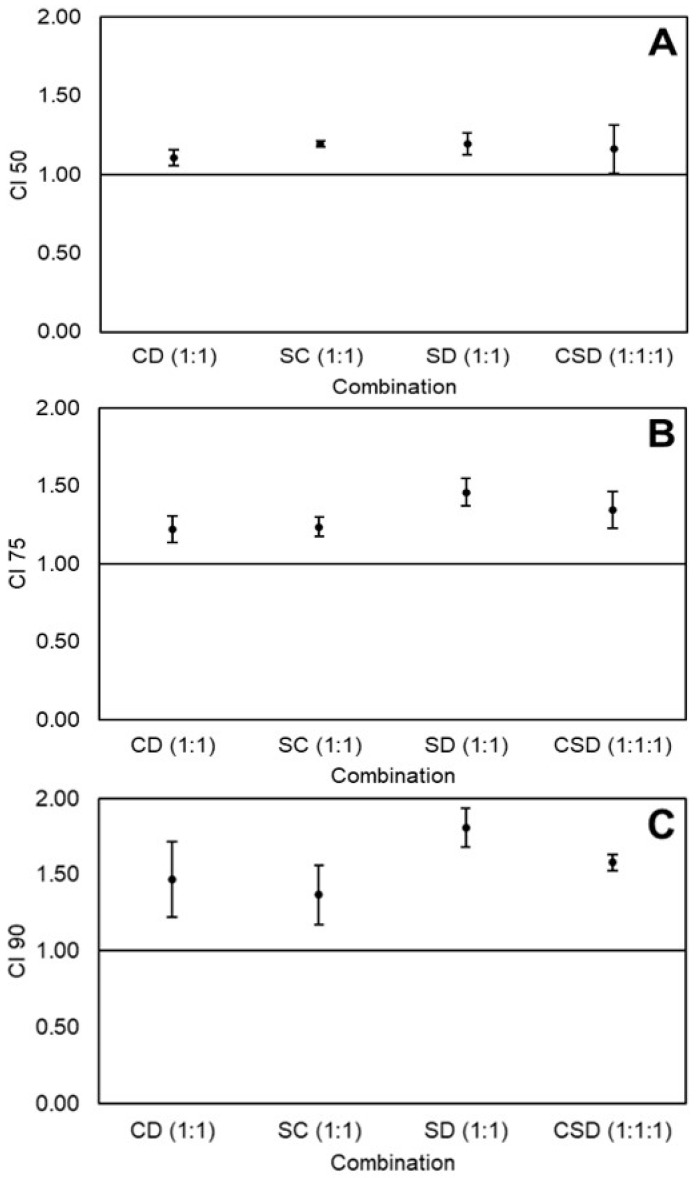
Combination index (CI) values of equimolar combinations of curcumin (C), sulforaphane (S), and dihydrocaffeic acid (D) on the viability of Caco-2 colon cancer cells at 50% (**A**), 75% (**B**), and 90% (**C**) cytotoxicity levels. Values represent the mean ± standard error of two independent experiments performed on different days, with each experiment having three replicates. Abbreviations: CI 50, 75, and 90 = Combination index at 50%, 75%, and 90% cytotoxicity.

The CI-values for the equimolar combinations of curcumin, sulforaphane, and dihydrocaffeic acid on the viability of Caco-2 cells at 50%, 75%, and 90% cytotoxicity level are illustrated in Figure 2A–C, respectively. All combinations were antagonistic at all cytotoxicity levels, and this antagonism tended to increase with increasing cytotoxicity.

### 2.4. Effects of Non-Equimolar Combinations of Curcumin, Sulforaphane, and Dihydrocaffeic Acid on the Viability of Caco-2 Cancer Cells

Since no synergistic combinations were detected in Caco-2 cells using equimolar combinations, a prescreening of different single-dose, non-equimolar combinations was performed (Table 3). The mixtures CD(1:4), CD(9:2), and CSD(7:1:1) showed synergy at relatively high cytotoxicity levels (50–65%). Hence, these combinations were thoroughly analyzed.

**Figure 3:**
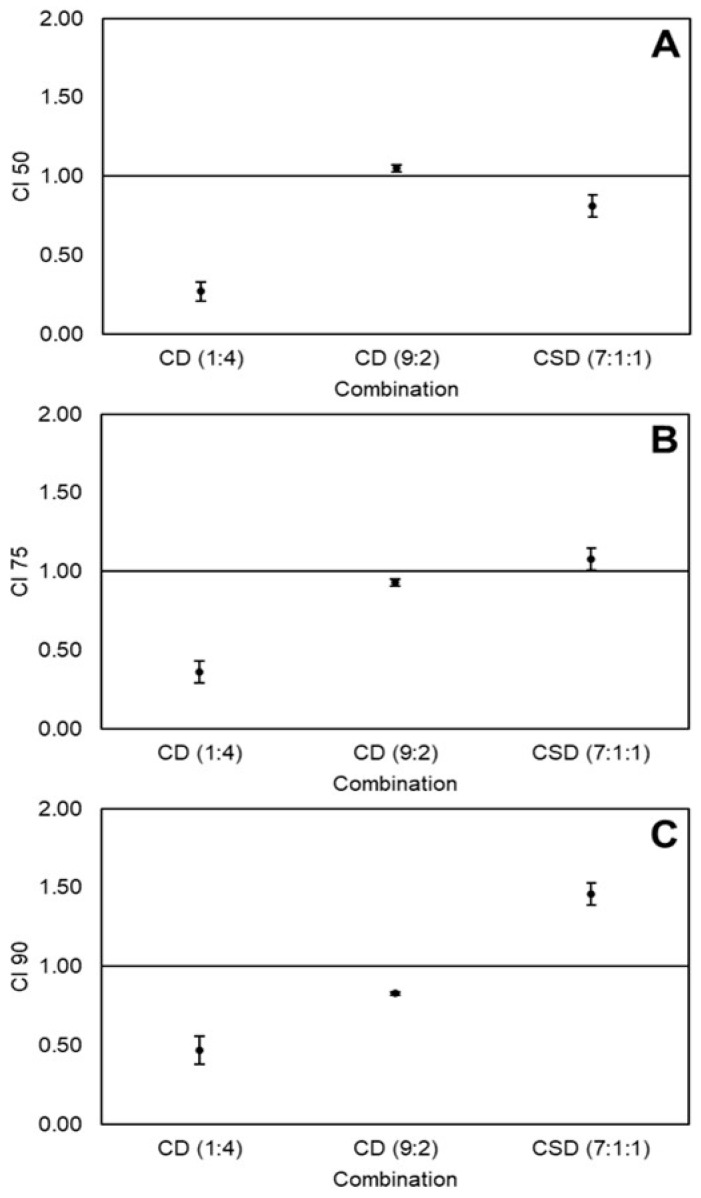
Combination index (CI) values of non-equimolar combinations of curcumin (C), sulforaphane (S), and dihydrocaffeic acid (D) on the viability of Caco-2 colon cancer cells at 50% (**A**), 75% (**B**), and 90% (**C**) cytotoxicity levels. Values represent the mean ± standard error of two independent experiments performed on different days, with each experiment having three replicates. Abbreviations: CI 50, 75, and 90 = Combination index at 50%, 75%, and 90% cytotoxicity.

Validated CI-values of combinations CD(1:4), CD(9:2), and CSD(7:1:1) at 50%, 75%, and 90% cytotoxicity levels are depicted in Figure 3A–C, respectively. CD(1:4) had a strong synergistic effect at all cytotoxicity levels (CI 0.25–0.5). Interestingly, the CD(9:2) combination showed nearly additive effects at 50% and 75% cytotoxicity levels, and synergistic effects at 90% cytotoxicity. Meanwhile, CSD(7:1:1) had an opposite tendency, starting at synergy at 50% cytotoxicity and moving to antagonism at 90% cytotoxicity. The doses of the individual nutraceuticals, as well as the doses and DRI values of the combinations at all cytotoxicity levels are shown in Table 4. The synergistic effects of CD(1:4) caused 4–7x dose reductions of both curcumin and dihydrocaffeic acid. On the other hand, CD(9:2) caused large dose reductions of dihydrocaffeic acid (17–25x) at all cytotoxicity levels; however, almost no dose reduction of curcumin was observed (DRI ≈ 1), explaining why most effects were additive. Likewise, although important dose reductions of sulforaphane (2.5–7x) and dihydrocaffeic acid (30–40x) were observed at all levels due to the CSD(7:1:1) combination, curcumin showed only small dose reductions at 50% and 75% cytotoxicity (DRI ≈ 1) and a small dose increase at 90% cytotoxicity (DRI < 1), explaining the tendency from synergy to antagonism.

### 2.5. Determination of Best Combination

**Figure 4:**
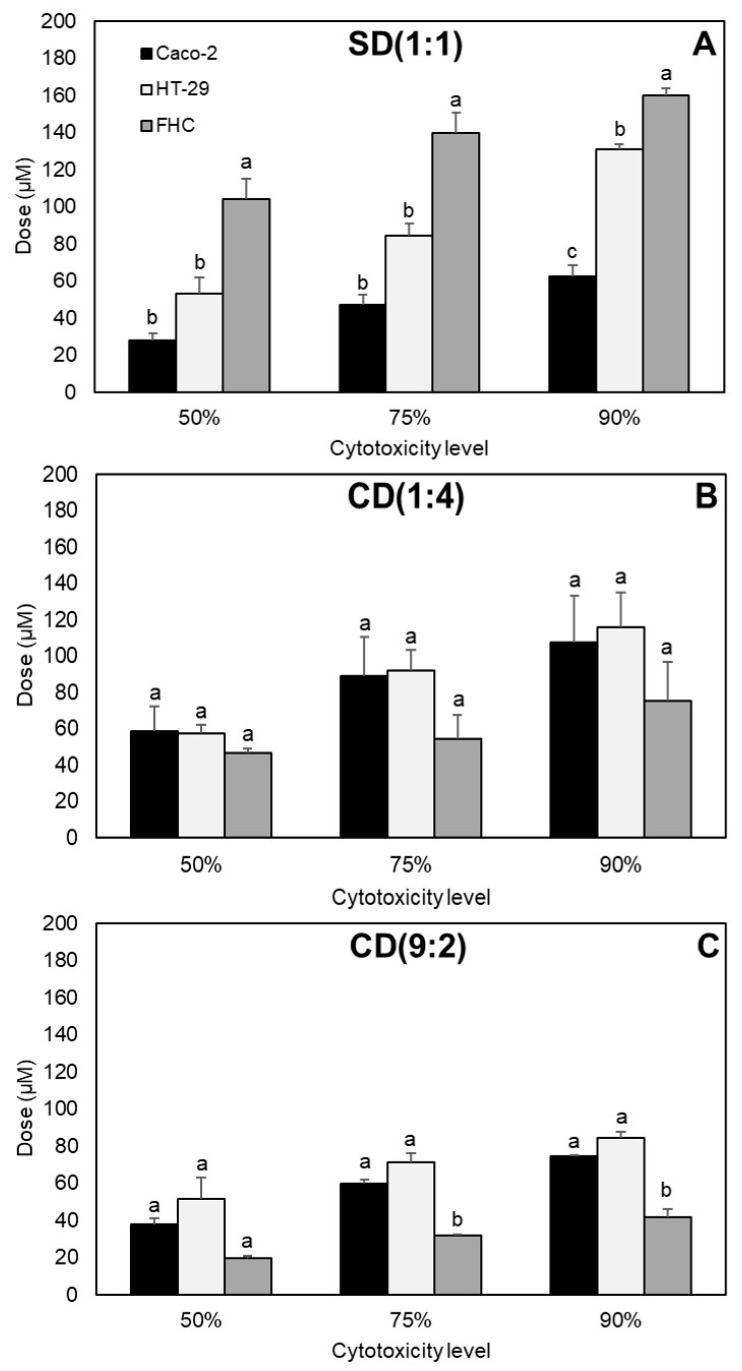
Cytotoxicity of synergistic combinations of curcumin (C), sulforaphane (S), and dihydrocaffeic acid (D) on colon cancer cells (HT-29 and Caco-2) and healthy colon cells (FHC). (**A**) SD(1:1), (**B**) CD(1:4), and (**C**) CD(9:2). Values represent the mean ± standard error of two independent experiments performed on different days, with each experiment having three replicates. Different letters in the same cytotoxicity level indicate statistically significant difference by Tukey’s test (*p* < 0.05).

The cytotoxicity of the synergistic combinations SD(1:1), CD(1:4), and CD(9:2) was compared between the colon cancer cells and healthy fetal human colon (FHC) cells at 50%, 75%, and 90% cytotoxicity levels (Figure 4A–C, respectively). SD(1:1) was significantly more cytotoxic for cancer cells than healthy cells at all cytotoxicity levels. No significant differences in cytotoxicity were found between the three cell lines with CD(1:4). Meanwhile, CD(9:2) was significantly more cytotoxic for healthy cells than cancer cells, particularly at high cytotoxicity levels (75% and 90%). Based on this information, SD(1:1) was chosen as the best combination.

## 3. Discussion

### 3.1. Some General Aspects about Cell Cycle and Apoptosis

Results herein demonstrated that the combination SD(1:1) was synergistic against HT-29 colon cancer cells at 90% cytotoxicity level (Figure 1), using relatively high doses (90:90 µM, Table 2). Likewise, the CD(1:4) combination was synergistic at all cytotoxicity levels (doses 9:36–34:136 µM, Table 4) and the combination CD(9:2) at 90% cytotoxicity level (doses 108:24 µM, Table 4) against Caco-2 colon cancer cells (Figure 3). Synergy can occur when two compounds cooperate on targets on the same or different pathways involved in the same process [2,38]. In order to understand the mechanisms through which SD(1:1), CD(1:4), and CD(9:2) exerted the synergistic effect against the viability of colon cancer cells, some aspects about the cell cycle and apoptosis need to be addressed. 

Many molecules and metabolic pathways are involved in cell cycle and apoptosis. Mitogen-activated protein kinases (MAPKs) belong to the superfamily of serine/threonine kinases, which play a central role in transducing various extracellular signals into the nuclei and regulate a variety of physiological processes, including cell growth, differentiation, cell cycle, and apoptosis. The extracellular-regulated kinase (ERK), c-jun N-terminal kinase (JNK), and p38 are the three most relevant MAPK pathways. The ERK pathway is generally believed to be prosurvival, while JNK and p38 are often considered as apoptotic [15]. Moreover, cyclins are a major group of proteins that control the cell cycle. Downregulation of cyclin D1 has been found to cause G_1_-phase cell-cycle arrest [39], while induction of cyclins A and B1 causes G_2_/M phase arrest [40]. In addition, it is known that p21 is an inhibitor of cell proliferation by restraining cyclin-dependent kinases (CDKs) [41]. Activation of the ERK and p38 pathways is involved in the upregulation of p21, whereas the activation of the JNK pathway is involved in the downregulation of cyclin D1 [39]. 

Furthermore, a major pathway through which apoptosis can be triggered is the mitochondria-dependent intrinsic pathway. Two main groups are involved in this pathway: the Bcl-2 family proteins and the caspase family. The Bcl-2 family is subdivided into three groups: the BH3-only proteins (e.g., Bid, Bad, Bik, Bim, Noxa, and Puma), the pro-apoptotic Bax subfamily (e.g., Bax, Bak, and Bok), and the anti-apoptotic Bcl-2 subfamily (e.g., Bcl-2 and Bcl-XL). On the other hand, caspases are cysteine aspartyl-specific proteases that regulate the initiation and final execution of apoptosis. In response to death stimuli (e.g., DNA damage), BH3-only proteins either activate the Bax members or antagonize the Bcl-2 members, which causes mitochondrial outer membrane permeabilization and consequently the release of cytochrome *c* into the cytoplasm. Cytochrome *c* then binds with apoptosis protease activating factor-1 (Apaf-1). Seven Apaf-1/cytochrome *c* complexes assemble to form the apoptosome in the presence of ATP/dATP. Then the apoptosome recruits inactive procaspase-9, facilitating its autocleavage into its active form caspase-9. Still bound to the apoptosome, caspase-9 subsequently activates caspase-3, and caspase-3 initiates a caspase cascade that leads finally to apoptosis [8,42,43,44].

Mitochondria have an outer membrane and an inner membrane, which are both key in the induction of apoptosis. The voltage-dependent anion channel (VDAC) is the most abundant protein of the outer membrane and is normally responsible for the transport of metabolites between the cytoplasm and the mitochondrial intermembrane space. Meanwhile, adenine nucleotide translocator (ANT) is the most abundant protein of the inner membrane and is a strictly specific antiporter that only exchanges ATP and ADP. When death stimuli activate proapoptotic proteins, such as Bax, they can interact with VDAC and ANT to form sufficiently large channels to release cytochrome *c* and initiate apoptosis. In addition, mitochondria are some of the intracellular components that produce reactive oxygen species (ROS) in a natural, controlled manner. However, when there is an excess of ROS (which can be caused, for instance, by cytotoxic agents), these species can interact directly with VDAC and ANT to facilitate pore opening and consequently cytochrome *c* release, ending in apoptosis [45].

### 3.2. Mechanisms of Sulforaphane and Dihydrocaffeic Acid against Colon Cancer Cells, and Proposed Model of SD(1:1) Synergy against HT-29

To fully understand how the combination SD(1:1) exerted a synergistic effect against HT-29 colon cancer cells, the different mechanisms of action of sulforaphane and dihydrocaffeic acid are described.

Previous reports have demonstrated that sulforaphane has several effects against HT-29. At a dose of 15 µM, sulforaphane caused cell-cycle arrest at G_2_/M phase and apoptosis in a dose-dependent manner in HT-29 [40]. Cell-cycle arrest was associated with an increase in the expression of cyclins A and B1, while apoptosis with an increase in proapoptotic protein Bax (but no effect on anti-apoptotic protein Bcl-2), and the release of cytochrome *c* from the mitochondria. Moreover, at high doses (>25 µM), sulforaphane dramatically induced in HT-29 the expression of p21 and inhibited the expression of cyclin D1, leading to cell-cycle arrest at G_1_ phase [39]. Additionally, the authors observed that sulforaphane activated several MAPK pathways, including ERK, JNK, and p38. They related all these effects with sulforaphane-induced oxidative stress. Since synergy was obtained at high doses of sulforaphane (90 µM) in our study, it is more likely that the mechanism of sulforaphane was similar to the one reported by Shen et al. [39]. 

Meanwhile, the anticancer mechanisms of dihydrocaffeic acid are unknown. It can be hypothesized, however, that the mechanisms are similar to caffeic acid, since they have almost identical chemical structures [27]. Jaganathan [46] reported an increase in ROS by caffeic acid in HCT 15 colon cancer cells, which caused a reduction in mitochondrial membrane potential and consequently apoptosis. Although it may seem paradoxical that an antioxidant such as caffeic acid can cause oxidative damage, several antioxidants follow a hormetic mechanism, where, at low concentrations, the compound can work as an antioxidant, but at high concentrations, it becomes prooxidant [47]. Dihydrocaffeic acid is mostly recognized as an antioxidant [48]. Hence, the mode of action of dihydrocaffeic acid could be a hormetic mechanism, where, at high doses, it causes oxidative damage, causing mitochondrial dysfunction and apoptosis. In fact, this explains why dihydrocaffeic acid required much higher doses, individually, to terminate colon cancer cells, compared with curcumin and sulforaphane (Table 2 and Table 4).

**Figure 5:**
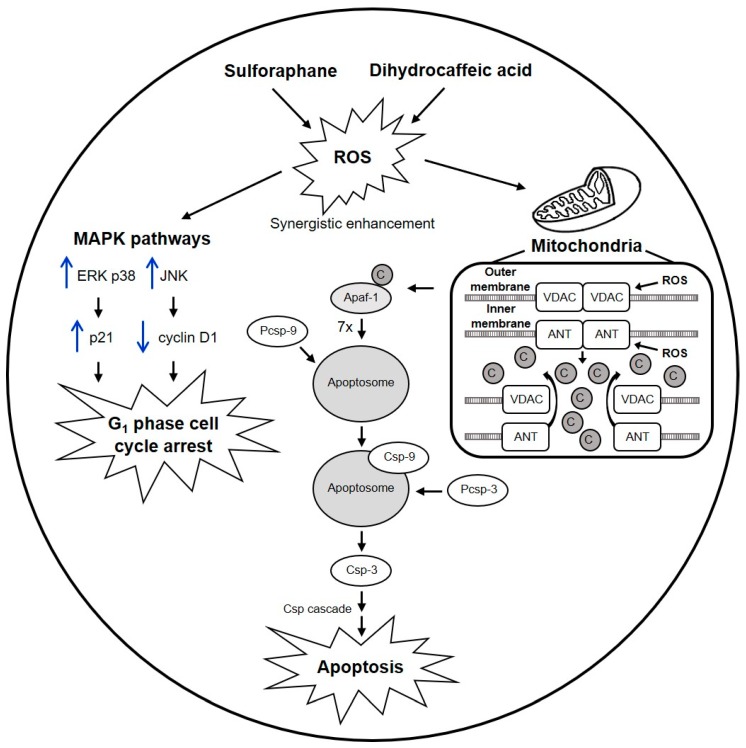
Hypothetical model of the synergistic mechanism of sulforaphane and dihydrocaffeic acid against the viability of HT-29 colon cancer cells. Black arrows represent metabolic pathways, whereas blue arrows indicate up- or downregulation of proteins. Sulforaphane and dihydrocaffeic acid work together to enhance reactive oxygen species (ROS) production to levels that cannot be reached by either compound alone. ROS activate the mitogen-activated protein kinase (MAPK) pathways extracellular-regulated kinase (ERK), c-jun N-terminal kinase (JNK), and p38. ERK and p38 upregulate p21, while JNK downregulates cyclin D1. The result of these events is cell-cycle arrest at G1 phase. Simultaneously, ROS attacks mitochondria, specifically the voltage-dependent anion channel (VDAC) and adenine nucleotide translocator (ANT) proteins, which are located in the outer and inner membranes, respectively. This facilitates pore opening, causing the release of cytochrome *c* (C) into the cytoplasm. Then, cytochrome *c* initiates the apoptotic intrinsic pathway, where (1) cytochrome *c* binds with apoptosis protease activating factor-1 (Apaf-1) to form a binary complex; (2) seven Apaf-1/cytochrome *c* complexes assemble to form the apoptosome; and (3) the apoptosome recruits inactive procaspase-9 (Pcsp-9), facilitating its autocleavage into its active form caspase-9 (Csp-9). Csp-9, still bound to the apoptosome, cleaves procaspase-3 (Pcsp-3) into its active form caspase-3 (Csp-3). Finally, Csp-3 initiates a caspase cascade (Csp cascade) that leads to apoptosis.

Since the combination SD(1:1) was chosen as the best (Section 2.5), we proposed a detailed model to explain the synergy of this combination against HT-29 based on the reported mechanisms (Figure 5). Briefly, sulforaphane and dihydrocaffeic acid synergistically enhanced ROS production. The high amount of ROS unleashed a chain of events, including activation of ERK, JNK, and p38 MAPK pathways, which in turn induced p21 and downregulated cyclin D1, causing cell-cycle arrest at G_1_ phase. Simultaneously, ROS acted on the VDAC and ANT proteins of the outer and inner mitochondria membranes, respectively, facilitating pore opening and subsequent cytochrome *c* release, resulting in the activation of the apoptotic intrinsic pathway.

### 3.3. Mode of Action of Curcumin against Colon Cancer Cells, and Proposed Mechanisms of CD(1:4) and CD(9:2) Synergy against Caco-2

Curcumin exerts several effects against colon cancer cell lines. Jaiswal et al. [6] reported induction of caspase-3-mediated β-catenin cleavage, degradation of E-cadherin and APC. Moreover, curcumin decreased transactivation of the β-catenin/Tcf-Lef complex and the promoter DNA-binding activity of the β-catenin/Tcf-Lef complex, downregulating c-myc and cdc2/cyclin B1 kinase activity in HCT-116 cells. These mechanisms were linked to alteration of the cell–cell adhesion pathway, p53- and p21-independent G_2_/M phase arrest, and apoptosis. Curcumin also caused apoptosis of HCT-116 by sustained phosphorylation and activation of the JNK pathway, induction of JNK-dependent phosphorylation of c-jun, expression of AP-1, and inhibition of constitutive NF-κB [49]. The importance of Bax in curcumin-induced apoptosis of HCT-116 was studied by Rashmi et al. [44]. Cells with one allele of Bax gene (Bax+/−) treated with curcumin showed decreased viability, activation of caspases 3 and 9, release of cytochrome *c*, and expression of second mitochondria derived activator of caspase (Smac) and apoptosis-inducing factor (AIF), whereas cells with Bax knockout (Bax−/−) were not affected. However, downregulation of the antiapoptotic protein Bcl-XL or overexpression of Smac caused apoptosis of Bax−/− cells after curcumin treatment, offering an alternative against Bax-deficient chemoresistant cancers. In addition, curcumin caused apoptosis in HT-29 cells by activating the expression of caspases 3 and 12, and furthermore through the involvement of calpain [8]. Finally, Goel et al. [50] found that curcumin inhibited cell growth of HT-29 through inhibition of cyclooxygenase-2 (COX-2). Hence, the synergistic effects of CD(1:4) and CD(9:2) against the viability of Caco-2 could be due to the multitarget anticancer activity of curcumin plus ROS generation by dihydrocaffeic acid, as proposed in Section 3.2.

### 3.4. Different Effects of Same Combinations between Colon Cancer Cell Lines

Different effects of the same combinations were observed when HT-29 and Caco-2 cells were compared. For instance, some of the equimolar combinations were additive or synergistic in HT-29 cells, whereas all equimolar combinations were antagonistic in Caco-2 cells (Figure 1 and Figure 2). Different responses to nutraceutical combinations between cancer cell lines could be due to differences in their genetic profiles, which may lead to differences in the metabolism rate of the compounds, expression of detoxifying enzymes, and efficacy of the multidrug resistance system to pump out cytotoxic compounds [47].

### 3.5. Different Effects of Varying Doses and Proportions of the Same Nutraceuticals in Colon Cancer Cells

Different doses and proportions of the same nutraceuticals caused different effects in HT-29 and Caco-2 cells. For instance, SD(1:1) caused an antagonistic effect at low doses (50% cytotoxicity level) but a synergistic effect at high doses (90% cytotoxicity level) in HT-29 cells (Figure 1). Meanwhile, CD(1:1) was antagonistic at all cytotoxicity levels in Caco-2 cells (Figure 2), but changing the ratio to CD(1:4) caused a synergistic effect at all studied cytotoxicity levels (Figure 3). A possible explanation could be that, at different doses and proportions, different mechanisms are activated [28]. This phenomenon has been observed with sulforaphane, where at low doses, it induced the expression of phase II detoxifying enzymes, while at high doses, it caused cell-cycle arrest and apoptosis in Caco-2 cells [51].

### 3.6. Selectivity of Synergistic Combinations between Healthy and Colon Cancer Cells

The cytotoxicity of the three synergistic combinations studied herein (SD(1:1), CD(1:4), and CD(9:2)) was compared between healthy (FHC) and colon cancer cells (HT-29 and Caco-2), to see if there was at least one combination that was more selective toward the malignant cells (Figure 4). The CD(1:4) and CD(9:2) combinations were similarly or even more cytotoxic for healthy cells than cancer cells (Figure 4B,C), suggesting that both mixtures attacked targets that were present in both healthy and cancer cells. Interestingly, the SD(1:1) combination was more selective toward both cancer cells than healthy cells at all cytotoxicity levels (Figure 4A). Sulforaphane has been reported to inhibit significantly more the cell proliferation of HCT-116 colon cancer cells than normal colon mucosa-derived (NCM460) cells, and to activate survival signaling in NCM460 and apoptotic signaling in HCT-116 cells [15]. In the case of oxidative stress, it is known that cancer cells consume higher amounts of oxygen than their healthy counterparts, in order to generate the necessary energy to upregulate multiple signaling pathways that promote proliferation, inhibit apoptosis, and promote migration and invasion of other tissues [47]. This comparatively higher oxygen consumption causes cancer cells to have a higher oxidative stress level than healthy cells. This important difference increases their survival potential, as larger amounts of ROS augment the probability of inducing mutations and redox signaling that inactivate apoptotic genes and activate prosurvival genes [52]. Therefore, the higher sensitivity of colon cancer cells to SD(1:1) could be attributed to a combination of the selectivity of sulforaphane toward colon cancer cells and a greater oxidative damage caused by an initial higher oxidative status plus ROS generated by sulforaphane and dihydrocaffeic acid.

## 4. Materials and Methods 

### 4.1. Materials

Curcumin, sulforaphane, dihydrocaffeic acid, dimethyl sulfoxide (DMSO), human recombinant epidermal growth factor (EGF), HEPES, cholera toxin, insulin, transferrin, and hydrocortisone were obtained from Merck (Kenilworth, NJ, USA). Dulbecco’s Modified Eagle Medium/Nutrient Mixture F-12 (DMEM/F12), McCoy’s 5A medium, human colon cancer cell lines Caco-2 (HTB-37) and HT-29 (HTB-38), and FHC healthy colon cells were purchased from American Type Culture Collection (ATCC, Manassas, VA, USA). Antibiotic–antimycotic (Anti–Anti) solution (10,000 U/mL penicillin, 10,000 µg/mL streptomycin, and 25 µg/mL amphotericin B) and fetal bovine serum (FBS) were acquired from Gibco Invitrogen (Carlsbad, CA, USA). CellTiter 96^®^ AQueous One Solution Proliferation Assay kit was purchased from Promega (Madison, WI, USA). 

### 4.2. Cell Culture

Caco-2 and HT-29 cells were grown in DMEM/F12 and McCoy’s media, respectively. FHC were grown in DMEM/F12 supplemented with 25 mM HEPES, 10 ng/mL cholera toxin, 0.005 mg/mL insulin, 0.005 mg/mL transferrin, 100 ng/mL hydrocortisone, and 20 ng/mL EGF. All media were supplemented with 10% FBS and adjusted to pH 7.4. Cells were maintained at 80–90% confluency in tissue culture Petri dishes and incubated in a humidified incubator at 37 °C and 5% CO_2_. Culture medium was changed every 3 days. 

### 4.3. Combination Studies

Curcumin, sulforaphane, and dihydrocaffeic acid were tested, individually and in different combinations, over the viability of Caco-2 and HT-29 cells. All possible combinations were evaluated, i.e., CD, SC, SD, and the triple combination CSD. To determine synergistic, additive, and antagonistic effects of the combinations, the CI method was used [37], which consists of the following equation:

(1) $${\left({\mathrm{CI}}_{x}\right)}_{n}=\overset{n}{\underset{j=1}{\sum }}\frac{{\left(D\right)}_{j}}{{\left(D_{x}\right)}_{j}}$$

where (CIx)n is the combination index for n compounds (in this case, nutraceuticals) that achieve effect x (in this case, % cytotoxicity), (D)j is the dose of each nutraceutical in the mixture that achieves effect x, and (Dx)j is the individual dose of each nutraceutical that produces the same effect x. The effects chosen were the cytotoxic concentrations to reduce the cell viability by 50%, 75%, and 90% (CC_50_, CC_75_, and CC_90_, respectively). The CI-value determines the type of effect. If CI < 1, =1, or >1, then the combination is considered synergistic, additive, or antagonistic, respectively.

The CompuSyn software (version 1.0) was used to process the dose-effect data. For all individual nutraceuticals and constant-ratio combinations used, 6-point dose-effect curves were constructed and linearized, using the following equation:

log(fa/fu) = m log(D) − m log(D_m_) (2)

where D = dose, fa = fraction of cells killed at dose D, fu = fraction of unaffected cells by dose D (fu = 1 − fa), D_m_ = CC_50_, and m = coefficient that indicates the form of the dose-effect curve (hyperbolic: m = 1, sigmoidal: m > 1, and flat sigmoidal: m < 1). The software was firstly used to validate that the data were good enough to be analyzed with the CI method. According to Chou [37], the data are valid if r > 0.95 for individual compounds and constant-ratio combinations in vitro, using Equation (2). Once the data were validated, the software was used to calculate the CI-values, the individual and combined doses, and the DRI (a measure of how many times the dose of a compound is reduced due to a combination) at CC_50_, CC_75_, and CC_90_.

To minimize the number of combinations that need to be tested for finding synergistic combinations, equimolar combinations were done first (1:1 or 1:1:1). If no synergistic combinations were found (i.e., no combinations gave CI < 1), then non-equimolar combinations were assayed. The latter was done by first performing a prescreening, using 6-point dose-effect curves of the individual compounds and three single-dose combinations at different non-equimolar proportions for each type of combination (CD, SC, SD, and CSD). The doses and proportions of each non-equimolar combination were selected based on the CC_50_ values of the individual compounds. Briefly, the individual dose ((Dx)j value of Equation (1)) of each nutraceutical was substituted with its corresponding CC_50_ value, and then the dose of each nutraceutical in combination ((D)j value) was chosen, using two criteria. (1) The (D)j values should give a CI < 1, as illustrated in Equation (3):

(3) $$\frac{D_{1}}{{\left({\mathrm{CC}}_{50}\right)}_{1}}+\frac{D_{2}}{{\left({\mathrm{CC}}_{50}\right)}_{2}}+\frac{D_{3}}{{\left({\mathrm{CC}}_{50}\right)}_{3}}<1$$

where (CC_50_)_1,2,3_ and D_1,2,3_ are the CC_50_ and the dose in combination of nutraceuticals 1, 2, and 3, respectively. (2) For each type of combination, there should be at least one non-equimolar combination where each of the nutraceuticals predominates (e.g., for combination type CD, there should be at least one combination where C predominates, and another combination where D predominates). The data were then analyzed as non-constant ratio combinations, using the same software, where it is required to use linearized curves of the individual compounds with r > 0.95 (Equation (2)), but allows the use of single-point data of the combinations, to determine the CI-value [37]. Those non-constant ratio combinations that were preliminarily synergistic were then validated by repeating the experiment as constant-ratio combinations, using 6-point curves, as previously described. All individual nutraceuticals and their combinations were tested on the same day. 

### 4.4. Cell Viability Assay

Cell viability was evaluated by using the 3-(4,5-dimethylthiazol-2-yl)-5-(3-carboxymethoxyphenyl)-2-(4-sulfophenyl)-2H-tetrazolium (MTS) assay. Cells were seeded in tissue culture 96-well microplates (15,000 cells/well) in 100 µL of medium supplemented with 1% Anti–Anti solution and incubated for 5 h at 37 °C and 5% CO_2_. All individual nutraceuticals and their combinations were dissolved in DMSO and serially diluted in medium supplemented with 1% Anti–Anti solution. Each dilution was added to the cells, at appropriate doses (µM), to obtain CC_50_, CC_75_, and CC_90_. For all cases, the final concentrations were 0.5% DMSO and 1% Anti–Anti. Cells with only medium and 0.5% DMSO were used as controls and considered 100% viable. A second control with only medium was used to verify that 0.5% DMSO was not cytotoxic to the cells, and indeed no cytotoxicity was observed (data not shown). Cells were incubated with each nutraceutical treatment for 24 h at the same culture conditions. After treatment, CellTiter 96^®^ was added (20 µL) and cells were incubated for 1 h. Absorbance was read at 490 nm in a microplate reader (Synergy MX, BioTek Instruments, Winooski, VT, USA). 

### 4.5. Determination of Best Combination

Cytotoxicity of all synergistic combinations was evaluated on Caco-2, HT-29, and FHC, using the same viability assay as described in Section 4.4. The combination that had more selectivity toward cancer cells was chosen as the best. 

### 4.6. Statistical Analysis

For all cases, two independent experiments were done on different days, and each experiment had three replicates. Results were expressed as means ± standard errors. Significant statistical differences were determined by One-Way Analysis of Variance (ANOVA), followed by Tukey’s test (*p* < 0.05), using Minitab 18 software (State College, PA, USA).

## 5. Conclusions

In summary, different combinations of curcumin, sulforaphane, and dihydrocaffeic acid were evaluated over the viability of human colon cancer cells. SD(1:1) was synergistic against HT-29 cells at 90% cytotoxicity level, while CD(1:4) was synergistic at all cytotoxic levels and CD(9:2) was synergistic at 90% cytotoxicity level against Caco-2 cells. The best combination was SD(1:1), since it was both synergistic and significantly more cytotoxic for colon cancer cells than healthy colon cells. Mechanistic studies are needed to validate the proposed model of synergy between sulforaphane and dihydrocaffeic acid. It would be interesting to also assess the type of effect the combinations SD(1:1), CD(1:4), and CD(9:2) have on normal colon cells. The SD(1:1) combination can serve as a basis for developing novel and highly effective food products for the prevention/co-treatment of colon cancer, using the proper ingredients and following an appropriate methodology.

## Figures and Tables

**Table 1:** R-values of linearized dose-effect curves of curcumin (C), sulforaphane (S), dihydrocaffeic acid (D), and their constant-ratio combinations.

Cell Line	Nutraceutical/Combination (Proportions)	r
HT-29	C	0.979 ± 0.003
	S	0.989 ± 0.002
	D	0.980 ± 0.010
	CD(1:1)	0.989 ± 0.004
	SC(1:1)	0.970 ± 0.008
	SD(1:1)	0.984 ± 0.014
	CSD(1:1:1)	0.981 ± 0.002
Caco-2	C	0.997 ± 0.001
	S	0.971 ± 0.026
	D	0.982 ± 0.009
	CD(1:4)	0.952 ± 0.004
	CD(9:2)	0.996 ± 0.001
	CSD(7:1:1)	0.986 ± 0.002

Values represent the mean ± standard error of two independent experiments performed on different days, with each experiment having three replicates. The equation to linearize the dose-effect curves was log(fa/fu) = m log(D) − m log(Dm) [37], where D = dose, fa = fraction of cells killed at dose D, fu = fraction of unaffected cells by dose D, Dm = CC_50_ (cytotoxic concentration to kill 50% of cells), and m = coefficient that indicates the form of the dose-effect curve.

**Table 2:** Doses and dose-reduction index (DRI) values of curcumin (C), sulforaphane (S), dihydrocaffeic acid (D), and their equimolar combinations at different cytotoxicity levels of HT-29 colon cancer cells.

Cytotoxicity Level		50%			75%			90%	
Compound	C	S	D	C	S	D	C	S	D
Individual dose (µM)	31.6 ± 4.2b	41.3 ± 14.6b	188.4 ± 9.4a	60.6 ± 6.0b	81.9 ± 29.4b	389.5 ± 34.7a	116.3 ± 7.5b	162.2 ± 59.1b	806.2 ± 103.5a
Combination dose (µM)									
CD(1:1)	33.9 ± 9.6b		33.9 ± 9.6b	59.0 ± 14.6b		59.0 ± 14.6b	102.6 ± 21.9b		102.6 ± 21.9b
SC(1:1)	46.5 ± 10.0b	46.5 ± 10.0b		96.5 ± 26.8b	96.5 ± 26.8b		200.7 ± 68.2b	200.7 ± 68.2b	
SD(1:1)		40.9 ± 4.7b	40.9 ± 4.7b		60.1 ± 11.5b	60.1 ± 11.5b		88.8 ± 23.5b	88.8 ± 23.5b
CSD(1:1:1)	19.4 ± 7.4b	19.4 ± 7.4b	19.4 ± 7.4b	32.3 ± 8.1b	32.3 ± 8.1b	32.3 ± 8.1b	55.0 ± 6.0b	55.0 ± 6.0b	55.0 ± 6.0b
DRI									
CD(1:1)	1.00 ± 0.2b		6.1 ± 2.0ab	1.1 ± 0.2b		7.2 ± 2.4ab	1.2 ± 0.2b		8.5 ± 2.8ab
SC(1:1)	0.7 ± 0.1b	0.9 ± 0.1b		0.7 ± 0.1b	0.8 ± 0.1b		0.6 ± 0.2b	0.80 ± 0.0b	
SD(1:1)		1.00 ± 0.2b	4.7 ± 0.8ab		1.3 ± 0.2b	6.8 ± 1.9ab		1.8 ± 0.2b	10.1 ± 3.8ab
CSD(1:1:1)	1.8 ± 0.5ab	2.2 ± 0.1ab	11.6 ± 4.9a	2.00 ± 0.3b	2.5 ± 0.3b	13.2 ± 4.4a	2.1 ± 0.1b	2.9 ± 0.8b	15.1 ± 3.5a

Values represent the mean ± standard error of two independent experiments performed on different days, with each experiment having three replicates. Different letters in the same cytotoxicity level (50%, 75%, or 90%) and quadrant (dose or DRI) indicate statistically significant difference by Tukey’s test (*p* < 0.05).

**Table 3:** Preliminary combination index (CI) values of non-equimolar combinations of curcumin (C), sulforaphane (S), and dihydrocaffeic acid (D) on the viability of Caco-2 colon cancer cells.

Combination	Doses (μM)	Proportions	Cytotoxicity Level (%)	CI
CD	45:10	9:2	64	**0.79 ± 0.01g**
	35:20	7:4	15	1.41 ± 0.36efg
	20:80	1:4	52	**0.88 ± 0.02fg**
SC	30:10	3:1	35	2.63 ± 0.09bc
	25:5	5:1	29	2.31 ± 0.15bcd
	10:35	2:7	34	1.58 ± 0.15ef
SD	30:10	3:1	25	2.90 ± 0.19b
	15:10	3:2	5	2.80 ± 0.40b
	15:80	3:16	44	1.66 ± 0.10de
CSD	35:5:5	7:1:1	62	**0.90 ± 0.07fg**
	15:5:80	3:1:16	5	3.70 ± 0.52a
	5:25:5	1:5:1	38	2.06 ± 0.05cde

Values represent the mean ± standard error of two independent experiments performed on different days, with each experiment having three replicates. Different letters indicate statistically significant difference by Tukey’s test (*p* < 0.05). Synergistic combinations are highlighted in bold.

**Table 4:** Doses and dose-reduction index (DRI) values of curcumin (C), sulforaphane (S), dihydrocaffeic acid (D), and their non-equimolar combinations at different cytotoxicity levels of Caco-2 colon cancer cells.

Cytotoxicity Level		50%			75%			90%	
Compound	C	S	D	C	S	D	C	S	D
Individual dose (µM)	65.8 ± 2.3b	44.6 ± 2.6bc	248.0 ± 11.7a	94.6 ± 2.7b	48.7 ± 2.5cd	386.8 ± 3.6a	135.9 ± 2.9bc	53.2 ± 2.5cde	605.3 ± 39.8a
Combination dose (µM)									
CD(1:1)	8.8 ± 2.2e		35.3 ± 8.8cde	17.2 ± 3.6de		68.7 ± 14.5bc	33.5 ± 5.8de		134.0 ± 23.2bc
SC(1:1)	65.4 ± 3.6b		14.5 ± 0.8de	83.6 ± 3.7b		18.6 ± 0.8de	106.9 ± 3.5bcd		23.8 ± 0.8de
CSD(1:1:1)	42.7 ± 5.6bcd	6.1 ± 0.8e	6.1 ± 0.8e	78.0 ± 7.9bc	11.1 ± 1.1e	11.1 ± 1.1e	142.6 ± 10.1b	20.4 ± 1.4e	20.4 ± 1.4e
DRI									
CD(1:1)	7.89 ± 1.71c		7.41 ± 1.52c	5.73 ± 1.06c		5.91 ± 1.30c	4.17 ± 0.63b		4.71 ± 1.11b
SC(1:1)	1.01 ± 0.02c		17.07 ± 0.15b	1.13 ± 0.02c		20.87 ± 1.12b	1.27 ± 0.01b		25.56 ± 2.51a
CSD(1:1:1)	1.56 ± 0.15c	7.37 ± 0.55c	41.07 ± 3.49a	1.22 ± 0.09c	4.39 ± 0.22c	35.09 ± 3.88a	0.96 ± 0.05b	2.62 ± 0.06b	30.00 ± 4.08a

Values represent the mean ± standard error of two independent experiments performed on different days, with each experiment having three replicates. Different letters in the same cytotoxicity level (50%, 75%, or 90%) and quadrant (dose or DRI) indicate statistically significant difference by Tukey’s test (*p* < 0.05).

## Abbreviations

ACF	Aberrant crypt foci
AIF	Apoptosis inducing factor
ANT	Adenine nucleotide translocator
Anti–Anti	Antibiotic–antimycotic
Apaf-1	Apoptosis protease activating factor-1
C	Curcumin
CC_50,75,90_	Cytotoxic concentration to reduce cell viability by 50%, 75%, and 90%, respectively
CI	Combination index
CDK	Cyclin-dependent kinase
COX-2	Cyclooxygenase-2
D	Dihydrocaffeic acid
DMEM/F12	Dulbecco’s Modified Eagle Medium/Nutrient Mixture F-12
DMSO	Dimethyl sulfoxide
DRI	Dose-reduction index
EGF	Epidermal growth factor
ERK	Extracellular-regulated kinase
FBS	Fetal bovine serum
FHC	Fetal human colon
JNK	c-jun N-terminal kinase
MAPK	Mitogen-activated protein kinase
MTS	3-(4,5-dimethylthiazol-2-yl)-5-(3-carboxymethoxyphenyl)-2-(4-sulfophenyl)-2H-tetrazolium
ROS	Reactive oxygen species
S	Sulforaphane
Smac	Second mitochondria derived activator of caspase
VDAC	Voltage-dependent anion channel
